# Emergency medicine in Nepal: present practice and direction for future

**DOI:** 10.1186/s12245-016-0118-3

**Published:** 2016-07-15

**Authors:** Nishant Raj Pandey

**Affiliations:** Department of Emergency Medicine, Kathmandu Model Hospital, Public Health Concern Trust, Pradarshani Marg, Kathmandu, Nepal

**Keywords:** Nepal, Emergency medicine, Emergency medical system, Emergency physicians, Curriculum, Training

## Abstract

Emergency medicine is one of the youngest recognized specialties in Nepal, and its growth in clinical practice and academic development has been challenging. In this paper, we reviewed the current state of emergency medicine in Nepal based on review of the literature, personal observations and experience, and interviews with many Nepali and foreign emergency physicians. Most hospitals in Nepal have adopted a multi-specialist approach, where emergency room physicians are primarily general practitioners/family physicians or house officers. As physicians are receiving their training via various pathways, national standards in training and certification have not been developed. As a result, the scope of practice for emergency physicians and the quality of care vary greatly among hospitals. Difficult working conditions, physician recruitment, compensation, and academic enrichment remain major challenges in the development of emergency medicine. For the sustainable development of this specialty, more international guidance and local leadership is needed to standardize the training curriculum, to provide adequate funding opportunities for academic development and to promote the overall development of the emergency care system.

## Review

### Introduction

Nepal is a small land locked country (total area 147,181 sq. km) located in South Asia between China and India. Ranking 191st out of 213 countries in terms of gross national income (GNI) per capita [1], it is estimated that nearly one fourth of Nepal’s 27.8 million people are living in poverty. The average expectancy of the Nepalese is 71, while compared to other south Asian countries, Sri Lankans live longer to be 76, Bhutanese at 69, Indians and Pakistanis are both at 66, and Afghanistan have the lowest life expectancy at 56 [2]. In terms of access to health services, the World Health Organization (WHO) has identified Nepal as one of 49 priority countries that have a critical shortage of health service providers. Estimates indicate that Nepal has a density of seven health service providers per 10,000 people, which falls well below the WHO-defined critical threshold of 23 per 10,000. Comparing to other south Asian countries, Nepal is better than Bangladesh, which has a density of six health service providers per 10,000 people, same as Afghanistan and a bit lower to Pakistan with 12 health service providers per 10,000 people. However, other south Asian counties like India, Sri Lanka, Maldives, and Bhutan do not fall into priority countries [3]. Nepal’s high rate of poverty and lack of health service provider coverage certainly contribute to the injury burden and to the lack of quality emergency care in Nepal, which was heavily felt during the recent natural disaster on April 2015. For a population of 27 million, Nepal has 743 hospitals, including both public (102) and private (641) ones. There are an additional 4000 primary healthcare centers (PHCC), health posts (HP), and sub-health posts (SHP) reaching into the rural areas. Most of the public hospitals are funded by the government, local communities, and local business and even by local religious organizations; however, all of the PHCC, HP, and SHP are solely funded by the government. It is estimated that about 70 % of people in Nepal utilized public health services in the fiscal year 2013/2014. However, to say that the emergency medical system in Nepal needs an upgrade would be an understatement. As all hospitals in the country fall under the jurisdiction of the Ministry of Health (MOH), and since there are no guidelines for management of emergency, trauma, or disaster preparedness set by the MOH, these are yet to be streamlined. In Table 1, levels of existing healthcare centers and their emergency care services have been summarized. Naturally, due to the country’s large rural population and its distribution over both mountainous and plain (Terai) regions, access to emergency care and the quality of such care remain significant challenges faced by the Nepalese healthcare system.

**Table 1:** Staffing in emergency departments in different levels of healthcare centers

Level of healthcare center	Service provider in emergency department
Primary: SHP^a^, HP^a^, PHCC^a^	Village health worker/auxiliary health worker/health assistant
	Medical officer/health assistant
Secondary: district hospitals, zonal hospitals, regional hospitals	Medical officers
Tertiary: central hospitals, teaching hospitals	Medical officers/general practitioners
Private hospitals	Medical officers/general practitioners

*SHP* sub-health post, *HP* health post, *PHCC* primary healthcare center

^a^Provide basic emergency care services with no designated emergency departments

Around 20 years ago, Meskin et al. [4] published one of the first formal evaluation of emergency care in Kathmandu. They concluded that there were 100,000 annual emergency department (ED) visits across three principal hospitals in Kathmandu. Focusing more specifically on emergency medicine (EM) in recent years, a retrospective observational study of ED visits at Kathmandu Medical College Teaching Hospital (KMC-TH) done over a 1-year period beginning in October 2002 reported that 9966 total patients were treated in the department [5]. A study from Patan Hospital states that each year it has approximately 250,000 outpatient visits and 30,000 ED visits [6]. According to the respective hospitals’ official websites, the emergency department at BP Koirala Institute of Health Sciences (BPKIHS) serves more than 40,000 patients a year [7] and Tribhuvan University Teaching Hospital (TUTH) treats 40,472 patients annually [8].

Rapid technologic, political, and economic changes have led to significant epidemiologic and demographic changes in the Nepalese population—for example, increase in the incidence of road traffic accidents as well as of chronic conditions such as coronary artery disease, chronic obstructive pulmonary disease, stroke, cancer, and diabetes [9]. According to the annual report published by the Department of Health Services in 2014, there were 87,726 reported cases of road traffic accidents in the country, which is a 5.3 % increase from 83,287 cases in 2013.

A trend of rapidly increasing number of road traffic accidents is occurring despite improvements in the road conditions [10]. Population changes due to a rising influx of foreign nationals, non-Nepalese residents, and Nepalese foreign graduates have demanded improvements in acute and emergency healthcare. As a result, there is a growing need for the development of a well-designed and responsive emergency care system in Nepal. On April 25, 2015, a powerful earthquake of 7.8Mw hit the heart of Nepal. The initial quake, together with strong aftershocks, resulted in about 10,000 deaths, with more than 23,000 people injured and about two million more displaced. The emergency medical system, virtually in its infancy in Nepal, was found wanting. Had it not been for the swift response from the major teaching hospitals in responding to an earthquake that was epicentered relatively near to them, the numbers would have looked far worse. On the other hand, rural areas completely lacking any pre-hospital and emergency care certainly led to excess morbidity and mortality. After this devastating natural disaster, there were a few things learned. One of them, definitely, was the need for EM training in the country.

In the last three decades, emergency medicine in Nepal has undergone a tremendous growth. However, it is still an area of medical care that requires further specialty definition and formalized training. There are different models of practice in existence. There is no structured or formalized listing of the practice models. Therefore, the scope of practice is variable. In public ED, the ED physician performs procedures ranging from lumbar punctures to endotracheal intubations. In private hospitals, the typical ED provider does not perform any complicated procedures and other specialists are expected to execute the necessary interventions. As patients presenting to the ED become increasingly complex, with undifferentiated chief complaints, it is becoming evident that specialized EM training is necessary.

### Current state of EM

#### Hospital-based emergency care overview

The oldest hospital in the country, Bir Hospital, was established in July 1889. In 1982, TUTH started its first training in general practice, and this program has been since providing emergency care physicians for the country mainly because there was no EM training in the country. Over the next few years, emergency departments quickly emerged in many hospitals across the nation. The organizational emergency care, as defined and established internationally, is certainly under-resourced and underdeveloped. Even in established centers, emergency care is performed by house medical officers (HMOs), who are recent graduates after four and a half years of medical school training and an additional year of internship. However, most centers in the country are run by health assistants with just 2 years of training without any formal training in emergency care. That is far below the training of a general practitioner or an emergency physician, who has 3 to 4 years of specialized training in addition to the four and a half year of medical school and 1 year of internship.

Various models of emergency medicine practice have been adopted in the country. Greater awareness by patients regarding their health, alongside of an economy growing in an environment of limited resources, have led hospitals within the same city to adopt different models of practice, emphasizing their available specialists. In Bansbari, a small town within Kathmandu valley, The National Institute of Neurological and Allied Sciences specializes in neurological emergencies, while the Shahid Gangalal National Heart Centre serves as the cardiac center and deals mainly with cardiac emergencies. Nepal still does not have a proper Pediatric ED staffed with pediatric ED physician. However, some of the pediatric hospitals provide emergency care but are staffed with pediatricians. Most of the patients arriving in these hospitals are referred from other smaller hospitals, where they are evaluated by local emergency personnel first.

Planning for streamlining of trauma management had begun as early as 1997, when a piece of land was allocated in the heart of the capital for establishment of a National Trauma Center, a hospital solely dedicated for the management of trauma. To be built with technical and financial aid from India, it was supposed to be the first of its kind in South Asia. But because of the political instability in the country, the construction of the hospital completed only in July 2009, and it was officially open for services only in 2015.

#### ED working conditions

Appropriately 80 % care centers in big cities have adopted a multi-specialist approach where emergency room physicians are primarily family physicians or house officers. Patients presenting with surgical- or subspecialty-related complaints are typically evaluated by on-call specialists after triage. On arrival, patients are triaged into different areas based on their chief complaints. Crowding patient population, increase in hospital stay, and increasing demand for outpatient urgent care services have all contributed to overcrowding and extended stays in the emergency department. Thus, the role of the Nepalese emergency physician has evolved over the years to include acute, inpatient, as well as critical care medicine. In most of EDs with a strong surgical presence, surgeons are able to perform emergent operations.

#### Emergency medical services

There is very little pre-hospital care, although Nepal did establish its first proper ambulance service a few years ago. Walker et al. [11] describe the development the Nepal Ambulance Service (NAS). After several years of extensive research, coordinated planning, systems development, and pre-hospital emergency care trainings, NAS began offering pre-hospital emergency medical services (EMS) in 2011. NAS also trains its paramedics and uses a base hospital dispatch system and a single three-digit number for the greater Kathmandu area. The most recent published information available indicates that as of December 2013, the NAS was facilitating an average of nine ambulance responses per day [11]. A prospective observational study examining the modes of transportation for patients presenting to the emergency department at Patan Hospital indicated that only 9.9 % of the patients had arrived by ambulance. The majority of patients had arrived by taxi (53.6 %), followed by bus (13.5 %) and private vehicle (11.4 %) [12]. Considering the municipality of Kathmandu has a population of 975,453 [13], one can assume that there is more than one emergency per day that requires pre-hospital emergency medical services. In most part of the country, private ambulances are the only sources of pre-hospital care. Most ambulances are owned by local hospitals, cab companies, or even funeral homes, and the ambulance usually charges patients according to distance covered which are usually similar or lower than local cab services. Many of the ambulances are not able to accommodate any medical equipment and carry no formal paramedics. Both the adult and pediatric patients are carried in these ambulances. In a mountainous country like Nepal, helicopter-based emergency medical services (HEMS) are a great asset. Many preventable deaths could be avoided with such service. Although Nepalese private airline companies provide helicopter rescue services in these areas, most of their vehicles are not armed with any medical personnel or equipment, which places the current effort far below its potential.

However, in the field of wilderness and mountain medicine, there has been active research and practice related to high altitude and mountain climbing activities. These efforts are truly of an international scale and involve academic medical centers specializing in remote, wilderness, and mountain medicine globally. Attempts have been made to train local doctors in mountain medicine. However, an academic course approved by a medical council is yet to be started. Clinics have been set up in various locations leading up to base camps of the mountains like Everest and Annapurna which mainly cater to the tourists and mountaineers. Medical teams from abroad often accompany tourist groups [14].

### EM physicians

Emergency physicians typically work 50 to 60 h per week. Some hospitals require attending physicians to be on-call in the evenings, but the majority of attending physicians cover the ED during normal business hours which is 8:00 am to 5:00 pm. After-hour patient care is primarily provided by HMOs and supervised by seniors who have received a minimum of 3 years of post-graduate clinical training. However, in other centers, emergency physicians work 8- to 12-h shifts around the clock. A HMO is the attending physician in the ED, who is responsible for the initial evaluation of the patient. HMOs, as mentioned earlier, are fresh medical graduates, with exposure to the emergency department being limited to 1 month during internship. Hospitals have a policy of either having an emergency physician or on-call consultants for the time beyond the business hours. As the on-call consultants are often only available on the phone, the working environment can be a challenge in itself. EDs are regularly overcrowded and under-staffed. The patients usually overflow into the hallways and, in some instances, to the hospital lobby area. The Nepalese are a very family- and friend-oriented society. It is very common for an entire family or group of friends to accompany the sick or the injured to the emergency department and to be actively involved in their care. This adds to the chaos and confusion in a busy ED. The challenge of providing customer-oriented care to such a large volume of patients and their associates as well as increasing threats of legal and social liability related to medical error has added to the already stressful working environment.

As an evolving new specialty in Nepal, EM is undervalued in most centers and suffers from lack of recognition and support from other departments and hospital administration. Emergency physicians are not viewed as “specialists” by the general public [15]. In addition, physicians practicing EM earn inadequate salaries as compared to their counterparts with similar training [15].

#### Training

Nepal has a formal accreditation process for graduate medical education. However, a standardized post-graduate EM training curriculum has not been established. The absence of a national certifying body for the EM specialty has led to variable training standards across programs. The process of post-graduate (residency) training in emergency medicine is highly complex in Nepal. In 1982, Nepal established its first post-graduate program in general practice at TUTH. It is a 3-year program, initially started in the Institute of Medicine (IOM), and later conducted in BPKIHS and the National Academy of Health Sciences (NAMS) [16]. The Nepal Medical Council (NMC), the governing body for medical doctors in Nepal, first recognized emergency medicine as a specialty in December of 2013, after they received their first two EM-trained physicians from abroad.

In line with the growing demand for better hospital and pre-hospital care, interest in EMS/HEMS and in EM expressed by physicians has been increasing. Few have received EM-specific training by traveling abroad and have been certified as emergency physicians by the NMC since 2013. As of 2015, there are only four physicians with post-graduate training in emergency medicine practicing in the country. These physicians work as regular emergency providers as well as helicopter physicians at the Kathmandu Model Hospital, the Grande International Hospital, and the National Medical College.

BPKIHS and Patan Hospital have developed a curriculum for 18-month EM fellowship programs to follow an accredited post-graduation training program. The first batch of EM fellows graduated in 2015, and there are only six physicians who have completed the fellowship. TUTH in Kathmandu is offering a 3-year sub-specialty program in EM leading to the title of Doctor of Emergency Medicine. This follows a 3-year residency in GP, pediatrics, Ob/Gyn, medicine, or surgery. The first batch graduated in 2015, and there are only two physicians with this DEM degree. In Table 2, various routes to emergency medicine certification have been summarized. There is no formal nursing education in EM or formal EMT or paramedic courses in Nepal.

**Table 2:** Three routes to emergency medicine certification can be summarized as follows

Program	Time	Place	Pre-requisite	Selection process
Residency in EM	3 years	Abroad	Undergraduate degree, i.e., MBBS-recognized NMC	Entrance examination and interview
Fellowship in EM	18 months	BPKIHS and Patan Hospital	1. Post-graduate degree recognized by NMC	Entrance examination and interview
			2. Must have NMC registration	
			3. Emergency department work experience of 1 year	
DM in EM	3 years	TUTH	3-year accredited post-graduation training	Entrance examination and interview

#### Recruitment and academic development

Limited reimbursements, heavy workloads, difficult working conditions, legal/social issues, and lack of recognition and respect from colleagues all contribute to the difficulty in recruiting physicians for the ED. For many physicians who work in an ED, emergency medicine was not their initial specialty of choice [15]. However, as recognition of the specialty of emergency medicine grows in Nepal, the number of graduating physicians interested in EM is increasing quickly. At present, the main topics of research in EM are observational studies in trauma, infectious diseases, child health, and nutrition [17]. Nepalese emergency physicians have accepted new technologies from developed countries, but a lack of population-based clinical studies and practice standards has limited the development of evidence-based practices in Nepal.

### Future directions

A nationally standardized residency training curriculum and certification process should be developed. Standardized training and certification ensures that core competencies are met for each physician trained to be an emergency physician. Monitored training and certification will contribute significantly to ensure high quality of patient care. Nepal should develop a standardized 3-year training curriculum in EM where each trainee participates in a standardized, multi-specialty curriculum, including rotations in medical and surgical specialties, pediatrics, obstetrics/gynecology, as well as medical and surgical critical care. The aim of this program should be to train a new generation of emergency physicians with a standardized skill set that can be adapted to any ED within the country. The curriculum could be based on similar principles as those of the Doctor of Medicine (emergency medicine) course or a fellowship in emergency medicine, but with a longer training duration.

The Doctor of Medicine (emergency medicine) course and fellowships in emergency medicine may themselves be a temporary solution. However, these courses require years of training and expenditures which do not seem to be practical in terms of a sustainable growth of the specialist. The question is whether such programs can be sustained due to their duration and cost. These courses follow a 3-year residency program in GP, pediatrics, obstetrics/gynecology, medicine, or surgery, all of which follow five and half years of undergraduate study. Tuition fee for undergraduate Nepalese students is high, ranging from US $32,000 to $40,000 [18]. A trainee then has to spend around US $22,000 to $40,000 [19] to complete their post-graduate training, and further investments are then needed both in terms of time and cost to complete the specialization in EM. Therefore, for the development of emergency medicine in Nepal, starting a 3-year training curriculum in EM seems to be more sustainable due to its duration and cost.

There are limited opportunities for certification courses for those interested in learning BLS and primary trauma care, but no standardization exists. For the development of Emergency Care in Nepal, we further need crash courses like ACLS, PALS, ALTS, PHTLS, and AWLS and so on. Therefore, such courses should also be introduced to the country. There are limited informative and evidence-based educational resources available to emergency physicians in Nepal. Several textbooks have been published by well-respected experts in the specialty, but no single reference text book has been produced. Most CME activities are sponsored by pharmaceutical or medical equipment companies. A systematic and up-to-date educational platform is critical for advancing emergency medicine as a specialty and for ensuring continued quality of patient care.

Physicians practicing EM are struggling to establish themselves as specialists. The most important current task for Nepal’s emergency physicians is to advocate for policies, programs, and funding that would support further development of the specialty. Advocacy should be formed through professional organizations like the American College of Emergency Physicians, the European Society for Emergency Medicine, or the Australasian College for Emergency Medicine. Such an organization is yet to be formed in Nepal in order to work with government agencies, hospital administrators, academic institutions, other medical professions, civic organization, and the general public.

As this new specialty develops in Nepal, it will continue to face new obstacles. With the development of evidence-based practice, new educational requirements will emerge. Advancement in research, education, and evidence-based clinical practice will all play a vital role in the development of the specialty. Developing and starting a residency program in emergency medicine should be the next step for the advancement of emergency medicine in Nepal.

## Conclusions

The country has numerous hurdles to overcome on the path of improvement of EM, including financial, political, cultural, and institutional obstacles. Nepal’s new constitution, signed only on September 20, 2015, guarantees the right to essential healthcare, funded by the State. This is a very critical political context, which can advocate the need of EM and pre-hospital care as part of that package of services, and they need to be financed by the State and absorbed by capable and trained providers. Financial limitations make the change difficult, as resources are needed to fund the establishment of an EM-focused faculty. This affects the design of an EM residency training program, which would be necessary for a modified curriculum. Locally and culturally sensitive education must be developed to improve emergency medicine in a sustainable fashion. Given limited resources, efficient management through improved triage, management protocols, and team management of patients could significantly improve care in Nepal’s EDs.

Difficult working conditions, lack of support from other specialties and hospital administrators, as well as rising healthcare costs have all slowed the development of EM in Nepal. While it is well recognized that the ability to provide care to acutely ill patients is essential to any healthcare system, Nepal needs to strengthen its emergency care through standardization of residency training and continuing medical education. Recent natural disasters, such as the earthquake, have reinforced the need for improvements in disaster preparedness and response.

## References

[1] GNI per capita ranking, atlas method and PPP based. http://data.worldbank.org/data catalog/GNI-per-capita-Atlas-and-PPP-table. Updated 2014. Accessed 10/01, 2014.

[2] The World Bank. Data: Nepal. http://data.worldbank.org/country/nepal. Updated 2015. Accessed February 1, 2015.

[3] Health workforce: achieving the health-related MDGs. It takes a workforce! http://www.who.int/hrh/workforce_mdgs/en/. Updated 2010. Accessed 10/01, 2014.

[4] Meskin S, Huyler F, Gupta SK, Berger L (1997). Delivery of emergency medical services in Kathmandu, Nepal. Ann Emerg Med.

[5] Dulal P, Khadka SB (2004). Victims of road traffic crashes attending the emergency department of Kathmandu medical college teaching hospital. Kathmandu Univ Med J (KUMJ).

[6] Murdoch DR, Woods CW, Zimmerman MD, Dull PM, Belbase RH, Keenan AJ, SCOTT RM, Basnyat B, Archibald LK, Reller LB. The etiology of febrile illness in adults presenting to Patan hospital in Kathmandu, Nepal. Am J Trop Med Hyg. 2004;70(6):670–5. 15211012

[7] Emergency Hospital Services. http://bpkihs.edu/hospital-services/emergency-hospital-services.html. Accessed September 24, 2015.

[8] T.U.Teaching Hospital. Http://www.iom.edu.np/?page_id=182. Accessed September 24, 2015.

[9] Bhandari GP (2014). State of non-communicable diseases in Nepal. BMC Public Health.

[10] Yadav NK (2011). Road traffic accident: an emerging epidemic. Health Prospect.

[11] Walker R, Auerbach PS, Kelley BV, Gongal R, Amsalem D, Mahadevan S (2014). Implementing an emergency medical services system in Kathmandu, Nepal: a model for “white coat diplomacy”. Wilderness Environ Med.

[12] Gongal R, Dhungana B, Regmi S, Nakarmi M, Yadav B (2009). Need of improvement in emergency medical service in urban cities. JNMA J Nepal Med Assoc.

[13] National Population and Housing Census 2011 (PDF). National Planning Commission Secretariat, Central Bureau of Statistics (CBS), Government of Nepal. 2012.

[14] Maeder MM, Basnyat B, Harris NS (2014). From Matterhorn to Mt Everest: empowering rescuers and improving medical care in Nepal. Wilderness Environ Med.

[15] Hayes BW, Shakya R. Career choices and what influences Nepali medical students and young doctors: a cross-sectional study. Hum Resour Health. 2013;11(1):1. 10.1186/1478-4491-11-5 PMC3599062 23394308

[16] Hayes BW, Butterworth K, Neupane B (2008). Nepal’s general practitioners—factors in their location of work. MEJFM.

[17] Simkhada PP, Baral YR, van Teijlingen ER (2010). Health and medical research in Nepal: a bibliometric review. Asia Pac J Public Health.

[18] Shankar PR (2011). Undergraduate medical education in Nepal: one size fits all?. J Educ Eval Health Prof.

[19] Fee Structure2014 Batch. http://www.kusms.edu.np/index.php/our-programs/postgraduate#fee-structure-2015-batch. Accessed September 24, 2015.

